# The Position of the Medical Examiner for Life Assurance.—II


**Published:** 1892-06-25

**Authors:** 


### The Practitioner's Mirror and Retrospect.

[*The Editor will be glad to receive offers of co-operation and contributions from members of the profession. All letters should be addressed to* The Editor, The Lodge, Porchester Square, London, W.]

### MEDICINE.

### THE POSITION OF THE MEDICAL EXAMINER FOR LIFE ASSURANCE.*—II.

(*Continued.*)

In our previous issue attention was drawn to some of the more important points that require careful investigation in estimating the expectancy of lives. Without going into details as regards particular occupations, the lecturer remarked that it was of very great importance to consider the occupation of even the perfectly healthy lives. Some offices decline to accept the lives of male publicans, and great care is needed in accepting any person employed in bars, &c. The Sheffield knifegrinder is unassurable; he very seldom lives beyond the age of 35. It is important to ascertain whether the grinding is wet or dry. There is no great danger in wet knife grinding, the danger of knife grinding being due to the fine particles of steel inhaled during the grinding process. Again, upholstering is more dangerous to life when the work is amongst the mattress makers and those who use mill-puff than amongst those who work with the less dusty horsehair. But there are other conditions in which the occupation becomes important, and in which the judgment of the examiner must take the place of statistical tables, *e.g.*, where the life is not altogether sound in family or personal history. Thus, a baker, with a family history of phthisis or bronchitis would be a more risky life than a plumber or painter with the same family history, the personal histories being the same, although the occupation of painting or plumbing is, on the average, more dangerous to life than a baker's. The increased risk to the baker arises from the fact that bakers are peculiarly liable to lung affections from their exposure to sudden changes of temperature and the dusty atmosphere in which they work. Lung weakness is, therefore, more likely to develop into actual disease in a baker than in a painter, whose chief danger is lead poisoning and gout, and in whom conversely a family history with kidney lesions would be more serious than the same in a baker. The consideration of the relations of trade to the history of the individual has gained in importance since it has been found that invalid lives may be safely accepted if adequately rated up. It has, in fact, been shown by Dr. Symes Thompson that invalid life assurance may be a gain instead of a loss to an office. And if it is desirable for an office to accept invalid lives, it is of far greater importance for the invalid candidates to be accepted; but these are just the cases in which every medical referee must feel considerable difficulty in arriving at a just appreciation of the extra risk to be met by rating up. Having made the examination and obtained all the necessary data regarding the proposer, the physician has to decide whether the life is a first-class life, and that there is no feature in the case that may be regarded as tending to shorten his normal expectancy of life. If, however, there is a reason to believe that his life will be shortened in consequence of any such bad feature, the assurer must be rated up accordingly. The medical examiner is expected to calculate the amount of loading for these invalid lives, for though the acceptance or rejection of any life rests with the medical board, the examining physician is generally invited to express his opinion on these points, and unless he is acquainted with the method of estimating and of meeting risks his advice and report are apt to be very misleading. A technical knowledge of insurance often leads one to seek full information on certain weak points in a case, the absence of which may induce the medical board to advise rejection, while the fuller knowledge may enable the board to accept the risk at ordinary rates or with some addition. It is the common experience of medical referees and actuaries that the medical examiners show by their reports that they have not the knowledge required for rating up, and that they will recommend the addition of two or three years only when the statements in their report clearly indicate that nothing under fifteen or twenty years would meet the extra risk. On the other hand, lives are just as often unduly rated from the exaggeration of relatively slight defects by the inexperienced examiner. Then again, even when the additional risk has been properly estimated by the physician, he must be careful in his method of "loading" the life. For instance, if the candidate is thirty years of age and it is considered that his life will be shorter than the average healthy man's life by ten years, we find by reference to the table that the normal expectation of life at age 30 is thirty-four years, and that it is only at forty-four that the expectation of life is ten years less than this. We must therefore rate him up by adding fourteen years to his life (the difference between thirty and forty-four), and make him pay the premium that a healthy man of forty-four years would pay in order to cover the extra risk. Without considering the matter, we might fall into the error of simply adding ten years to make up for the risk in this particular instance.

We may very briefly allude to certain classes of disease which require more especial consideration in connection with life assurance.

Perhaps the most important of all are pulmonary disease, especially phthisis. It is scarcely necessary to say that any suspicion of actual phthisis in a candidate places him outside the range of acceptable invalid lives. The questions we have to consider are, has the candidate ever shown any tendency to lung disease himself, and how far the family history spoils his chances of normal length of life. As regards family history, the nature and character of hereditary influence must be considered on three points—firstly, the preceding generations; secondly, the collateral generations; thirdly, the descendants of the individual. Now the great majority of candidates for insurance have reached the age of thirty, that is to say, they have passed the period of life (fifteen to twenty) in which most consumptives begin to show signs of hereditary consumptive taint. Therefore, a history of phthisis in a parent would, to a certain extent, lose weight if the individual were himself physically sound, and the more so if he was born before the parent became consumptive. We must, however, beware of vague terms, as undoubtedly a large number of consumptives acquire the disease late in life, between the ages of fifty and eighty; these, however, are mainly due to health broken down from causes other than hereditary taint. Hereditary tendency to phthisis has generally been over-rated in the past. The import of a phthisical tendency in descendants is even less than in preceding generations. The most decisive evidence in family history is that of collaterals, especially brothers and sisters, for it often indicates the critical period in which it is likely to develop, and which, if safely passed, places the candidate in a better position. Moreover a phthisical taint often appears *de novo* in a family, the result of adverse influences, in invalid, aged, or alcoholic parents at the time of conception. The following rules, extracted from Minet's work on "Life Insurance," summarises the points at issue as well as it is possible to do:

* Address to Bristol Insurance Institute, by Watson Williams, M.D.

(1) One consumptive death in the immediate family of father, mother, brother, or sister. No positive personal objection. Fair average. No extra premium (*i.e.*, provided the proposer is some years above the age at which father or mother died young of phthisis; or is near the age of father or mother at death, if they are advanced in years. Otherwise an extra premium. (2) Two consumptive deaths in a numerous family, one parent especially; father above forty; one brother or sister. Personal points favourable. Age above that at which brother or sister died. Fair average. No extra premium. (3) Two consumptive deaths among brothers or sisters; good personal history. Extra according to age, but should not be accepted unless at or above the age when they died, and personal points good. (4) Two consumptive deaths, or both parents; good personal points. Extra according to age, and ages of parents at death. (5) Three deaths or more from consumption, as one parent and two brothers or sisters; personal points good; not to be taken unless above the critical period of the family, and only with heavy extra personal points not favourable, unassurable. (6) No deaths from phthisis in the immediate family, but personal tendency to these diseases shown by bronchitis, hæmoptysis, narrow chest, general configuration. Extra premium. (7) No death from phthisis; one from superficial cause; infant mortality of family great; survivors very young. Extra premium.

These rules are based on careful investigation of actual results in life assurance, and it is only by utilising such technical information that an examiner, no matter how cultured he may be in the science of medicine, can do justice to the proposer's claims.

*(To be continued.)*

